# Morale in Old Age and Its Association with Sociodemographic, Social, and Health-Related Factors in Different Age Groups

**DOI:** 10.1155/2020/3939718

**Published:** 2020-08-01

**Authors:** Marina Näsman, Johan Niklasson, Jan Saarela, Mikael Nygård, Birgitta Olofsson, Yngve Gustafson, Fredrica Nyqvist

**Affiliations:** ^1^Faculty of Education and Welfare Studies, Social Policy Unit, Åbo Akademi University, PB311 FI-65101, Vaasa, Finland; ^2^Department of Community Medicine and Rehabilitation, Geriatric Medicine, Sunderby Research Unit, Umeå University, 901 87 Umeå, Sweden; ^3^Faculty of Education and Welfare Studies, Demography Unit, Åbo Akademi University, PB 311 FI-65101, Vaasa, Finland; ^4^Department of Nursing, Umeå University, 901 87 Umeå, Sweden; ^5^Department of Community Medicine and Rehabilitation, Geriatric Medicine, Umeå University, 901 87 Umeå, Sweden

## Abstract

Morale can be viewed as a future-oriented optimism or pessimism regarding challenges associated with aging and is closely related to subjective well-being. Promoting morale in old age could be considered to have important implications for aging well, and increased knowledge about morale in different stages of old age is needed. Hence, the aim of this study was to investigate factors associated with morale in different age groups among old people. Data were derived from a survey conducted in 2016, as a part of the Gerontological Regional Database (GERDA). The sample consisted of 9,047 individuals aged between 65 and 86 years from Ostrobothnia and Southern Ostrobothnia in Finland, and Västerbotten in Sweden. Morale was measured with the Philadelphia Geriatric Center Morale Scale (PGCMS) and regressed upon a number of sociodemographic, social, and health-related factors using linear regression analyses. The results showed that older age was an independent factor explaining lower level of morale. Additionally, the sociodemographic, social, and health-related variables could explain a large proportion of the variance in morale. Perceived loneliness, having gone through a crisis in life, poor self-rated health, and depression were associated with lower morale, and sleeping well with higher morale, in all age groups. Furthermore, the oldest age groups seem to be more exposed to several risk factors of lower morale identified in this study. Multidimensional interventions targeting especially social and mental health and the oldest-old could therefore be recommended.

## 1. Introduction

In this study, we explore morale in old age, described as a multidimensional concept containing future-oriented optimism or pessimism regarding challenges associated with aging, satisfaction with oneself, a feeling that there is a place in the environment for oneself, and acceptance of things one cannot change [1, 2]. Furthermore, morale can be viewed as a cognitive dimension of subjective well-being (SWB) [3], while also containing some psychological and social aspects [1]. High morale has previously shown to have some protective features, such as increased survival and lower risk of developing depressive disorders [4, 5], suggesting that identifying risk factors as well as promoting factors for morale in old age is essential.

By large, SWB has progressively received increased attention from researchers as well as policy makers and can serve as an important marker of societal progress [6]. SWB can also be considered an essential part of successful aging [7], which has been a central policy aim following the challenge of an aging population. SWB is often divided into cognitive and affective components (e.g., [8]), although there is no clear-cut definition of SWB nor of its construct. In addition to morale, different concepts such as psychological well-being, life satisfaction, and quality of life have often been used synonymously with SWB. These measures often overlap but can still be considered distinct concepts with differing focus and meanings. To clarify, morale is thus in this study considered a distinct concept, although related to SWB, and is believed to have important implications for aging well. However, the effect of age on level of morale, and whether the associations vary by age, is unclear and has not been extensively studied. Hence, the aim of this study was to examine morale in different age groups among old people.

Various social and health-related factors such as social support, chronic conditions, and self-rated health have previously been associated with level of morale in old age (see, for example, [9–12]). Morale has also been associated with sociodemographic factors such as gender [13], level of education [13], income [11], and type of housing [14, 15]. In a recent five-year follow-up study of very old adults, the death of one's child, depressive disorders, and perceived loneliness emerging over the follow-up period were identified as key risk factors of a decrease in morale [16].

The amount of studies exploring age differences in morale is limited, and no study has to our knowledge had age as a main focus of inquiry. On the one hand, previous studies have indicated that the majority of very old adults have moderate or high morale [15, 17]. On the other hand, the study of Näsman et al. [17] showed also that 30.1 percent had on an individual level a significant decrease in morale over five years, indicating that a considerable proportion of the oldest-old is at risk of having lower morale over time. However, the level of morale did not differ significantly between the age groups (85, 90, and ≥95 years). In a cross-sectional study conducted by Woo et al. [18], morale was higher in those aged 90 and over compared to younger old. The study by Iwasa et al. [13] also showed that the level of morale in men, but not in women, was higher in older ages. The age span in their study was, however, 50–74 and thus did not include very old adults. The results of de Guzman et al. [12] showed on the contrary that older age was associated with lower morale (age span 65 to 85+). Prior information is thus not only scarce but also inconsistent.

Research on other measures of SWB, such as life satisfaction, has also come to somewhat conflicting results regarding age differences. There are studies claiming that well-being is u-shaped over the life span, i.e., higher in younger age and older age (e.g., [19]). There are, however, also studies indicating that well-being declines in very old age [20–22]. In a study of Jivraj et al. [23], SWB was higher in the older cohorts, but on an individual level, SWB decreased over time. Additionally, Gerstorf et al. [24] showed that decline in life satisfaction was more closely associated with distance to death than chronological age, suggesting that late-life changes in well-being are associated with terminal decline. It is further suggested that some dimensions of SWB remain stable into very old age, while others decline [22]. In the study by Hansen and Slagsvold [22], life satisfaction decreased over a five-year period, negative affect increased, and positive affect remained stable in the oldest age group (aged 75–79 at baseline). In contrast, Smith et al. [25] found a decrease in life satisfaction and positive affect but no increase in negative affect. Considering these inconsistent results, the question regarding whether level of morale varies in different age groups from younger old to very old age is still open.

Furthermore, it is also uncertain whether the associations between morale and different sociodemographic, social, and health-related variables varies in different stages of old age, and only one study on morale has so far touched upon this matter. In the study of Näsman et al. [16], no interaction effects between age and the variables included were found. However, the study included only individuals aged 85 years and older, so no comparisons with younger old were made, thus warranting further exploration.

To explore morale in different age groups, various social and health-related changes occurring in old age need to be taken into account. A certain change in the individual's social life is likely, which in turn can affect the well-being of the individual. Socioemotional selectivity theory (SST) is a lifespan theory of motivation and time perspective that offers explanations to these changes (e.g., [26]). It argues that, the time perspective, i.e., the perception of how much time you have left, rather than age in itself drives these changes [26, 27]. Changes in time perspective can in turn affect the individual's motivation and goal selection [26]. The changes in goal selection appear mainly in terms of acquisition of knowledge and emotion regulation. According to Carstensen et al. [28], people start to care more about experiencing meaningful social ties and less about expanding their horizons when they are approaching the end of life. This leads in turn to a greater investment in the quality of important social relationships and a generally enhanced appreciation of life. Accordingly, older adults' social networks include less peripheral contacts but equally many close social contacts as younger adults' [29]. SST adopts hence a quite positive outlook on aging in the sense that older adults, according to the theory, tend to use social selection and other types of social regulation as means to maintain high levels of well-being [30]. Consequently, close social ties could become increasingly important to morale as people reach advanced ages. Nevertheless, Charles and Carstensen [30] state that age-related advantages appear to be compromised if the individual is exposed to prolonged and unavoidable stress, stemming from, for example, various negative life events. It could hence be hypothesized that social network losses and restraints in health associated with very old age attenuate possible positive effects of aging mentioned in the SST and could also be expected to affect morale.

Very old age is often characterized as a period of frailty and high disease-burden and is related to the transition from the third age to the fourth age [31], which in turn can place considerable constraints on the well-being of the individual [25]. In a study of Puvill et al. [32], however, lower life satisfaction in those 85 years and older was mainly associated with poor mental health, in terms of depressive symptoms and loneliness, rather than poor physical health, which is similar to the results of Näsman et al. [16]. Furthermore, the gap between objective health and perceived well-being seems to be widened in advanced aging, suggesting that very old individuals can express a high sense of well-being even with deteriorated health, also known as the well-being paradox [33]. Accordingly, it could be expected that health predicts morale to a lesser extent in very old age compared to younger old age.

The selective optimization with compensation (SOC) theory, offers an explanation as to why very old adults are able to retain a high level of well-being despite adversities [34]. In short, the SOC theory entails an aspiration of maximizing gains and minimizing losses by using the strategies selection, optimization, and compensation (ibid.). In a recent study using the SOC theory as a starting point, a group who had high well-being despite low physical function was identified [35]. This group used the SOC-strategies to a higher extent than other groups included in the study. There were also individuals with relatively high physical functioning who still reported low well-being and who used SOC-strategies to a very limited extent, as well as those with low physical functioning who reported low well-being despite the use of SOC-strategies. The relationship between health and perceived well-being is thus highly complex, and to what extent health-related variables affect morale in different age groups needs to be further explored.

In sum, few studies have investigated age differences in morale in old age, thus motivating further examination. There is also a need for increased knowledge about whether the association between various sociodemographic, social, and health-related variables, and morale varies in different age groups, which could in turn have implications for policy as well as social and health-care development. Hence, the aim of this study was to examine factors associated with morale in different age groups among old people. In particular, we explored whether (1) the level of morale differed between the age groups, (2) whether possible differences could be explained by sociodemographic, social, and health-related factors, and (3) whether the same factors could explain level of morale in the different age groups.

## 2. Materials and Methods

### 2.1. Sample

The study is based on data from the GERDA (Gerontological Regional Database) survey collected in 2016 in Västerbotten, Sweden, and in Ostrobothnia and Southern Ostrobothnia, Finland. The aim of the multidisciplinary GERDA project is to investigate health and living conditions of older adults living in these regions, and it has been conducted in a collaboration between researchers from Umeå University in Sweden and Åbo Akademi University, Novia University of Applied Sciences, and Seinäjoki University of Applied Sciences in Finland. The participants were selected from the National Tax Board in Sweden and the Population Register Center in Finland. The questionnaire was sent out by post to every 66-, 71-, 76-, 81-, and 86-year-old (born in 1950, 1945, 1940, 1935, and 1930) living in the rural areas and in the city of Seinäjoki (Finland), whilst to every second living in the city of Vaasa (Finland), and every third in the city of Umeå and in the city of Skellefteå (Sweden). The questionnaire was sent out to 14,805 persons of whom 9,386 answered the questionnaire. In Finland, which is a bilingual country, the questionnaires were sent out in either Swedish or Finnish depending on the registered language of the respondent. The response rates and number of individuals in the different language groups/regions and age groups are presented in Table 1. The sample in the present study included 9,047 individuals (96.4% of the total sample) who fulfilled the inclusion criteria of answering 12 items or more in the Philadelphia Geriatric Center Morale Scale (PGCMS).

### 2.2. Measurements

Morale was assessed using the Philadelphia Geriatric Center Morale Scale (PGCMS) [36]. The instrument contains 17 questions with the answer alternatives “yes” and “no.” The scale has a maximum of 17 points, where each answer indicating high morale gives one point. In accordance with the scale instructions [37], unanswered questions were given zero points. The psychometric properties of the Swedish version of the instrument have been found satisfactory [38]. In line with previous studies [5, 16, 17], respondents were included if they had answered 12 items or more in the PGCMS. The distribution of the variable was somewhat negatively skewed (−0.973) but was due to the large sample size considered to meet the criteria for normality appropriately (e.g., [39]). Based on previous research on morale and the theoretical framework, a set of sociodemographic, social, and health-related variables were included, presented more in detail below.

#### 2.2.1. Sociodemographic Variables

The sociodemographic variables included information regarding education, marital status, and other living conditions. Ten years or more of education indicated higher educational level. Being in a relationship meant being married, cohabiting or living apart with a partner in opposite to being divorced, unmarried, or widowed. A respondent was considered to make ends meet without difficulties if he or she answered “without difficulties” as opposed to “with some difficulties,” “rather difficult,” and “very difficult” to the question “Do you make ends meet.”

Some previous research has shown that there are differences regarding health-related aspects such as self-rated health and psychological health between the language groups in Finland (e.g., [40]), as well as between Ostrobothnia and Västerbotten (e.g., [41]). The sample was therefore divided into three population groups in the variable “Region” (Västerbotten, Swedish-speakers in Ostrobothnia, and Finnish-speakers in Ostrobothnia and Southern Ostrobothnia). The sample was also divided into three groups in the variable “Place of residence” according to whether they had reported that they lived in an urban, semiurban, or rural area, since living in an urban or a rural environment has previously shown to affect mental well-being in older adults [42] but has to our knowledge not been previously investigated regarding morale. The variable “Born in the same place as you live now” was based on the question “Are you born/raised in the place where you live today?” (1 = yes, 0 = no).

#### 2.2.2. Social Variables

Frequency of social contacts was based on the question “How often do you have contact with the following persons?”. Friends and neighbors were merged into one variable, and children and grandchildren into another. A person was considered to have frequent contacts if he or she chose the alternative “several times a week” as opposed to “several times a month,” “sometime a year,” “never,” or “the person does not exist.” Membership in associations was dichotomized in the way that being an active member in at least one association was given the value one and not being a member, or being a passive member, was given the value zero.

The number of confidants was based on the question “Do you have a confidant with whom you can speak about anything that is sharing both concerns and joys?”. The answer alternatives included “spouse,” “children,” “grandchildren,” “siblings,” “parents,” “other relatives,” “friends,” “neighbors,” “home-care staff,” “nurses,” and “someone else.” The variable was dichotomized using median split (0-1 confidants = 0, 2 confidants or more = 1).

A person was considered to have high trust in friends and neighbors if the person chose the answer alternative “high trust” as opposed to “neither high or low trust,” “low trust,” or “cannot take a stand” to the question “How high trust do you have for the following persons and groups?”. In order to be considered to have high trust, the respondent needed to have high trust for at least one of the categories. Perceived loneliness was based on the question “Do you suffer from loneliness?” (1 = yes, 0 = no).

The variable “Gone through a crisis in life during the preceding year” was based on the question “Have you during the preceding year (12 months) experienced anything that you would consider a crisis in life?”. The alternatives were “yes”: “own disease,” “disease of a significant other,” “death of a family member,” “death of a friend,” “divorce,” “other problems in the family,” “moving to another location,” “worsening economy,” and “others” or “no: there have not occurred those kinds of changes” (0 = no crisis, 1 = one crisis or more). Since the majority of the events included in the variable could be considered interpersonal events, the variable was placed among the social variables.

#### 2.2.3. Health-Related Variables

Impaired vision was considered present if the respondent reported that he or she was unable to read the text in the newspaper with or without visual aids. Impaired hearing was considered present if the respondent stated that he or she was unable to hear normal conversation from a one-meter distance with or without hearing aids. The occurrence of stroke was based on the question “Have you had a stroke?” (1 = yes, 0 = no) and pain on the question “Have you had ache/pain during last week?” (1 = yes, 0 = no). Sleep was measured with the question “Do you have good night sleep”? (1 = yes, 0 = no/do not know) and having mainly own teeth with the question “Do you mainly have your own teeth?” (1 = yes, 0 = no).

The variable regarding instrumental activities of daily living (IADL) was based on four questions: “Do you clean your dwelling (vacuum and wipe the floor) without the help from others?”; “Do you do grocery shopping without the help from others?”; “Do you use public transportation such as busses, planes, or trains without the help from others?”; “Do you cook without the help from others?”. A person was considered independent in IADL if he or she answered yes to all four questions. The autonomy for personal activities of daily living (PADL) was measured with the question “Do you shower without the help from another person?”. If the respondent answered yes, he or she was considered independent in PADL. The question originates from the Katz ADL index [43], where bathing is listed as a measure of the least severe degree of disability.

Self-rated health was based on the SF-36 item “In general, would you say your health is:” with the answer alternatives “excellent,” “very good,” “good,” “fair,” and “poor” [44]. Answering fair or poor was regarded as having poor self-rated health. The variable “Depression” was based on the Geriatric Depression Scale with four items (GDS-4) and the question “Do you feel depressed?”. Depression was deemed present if the respondent had answered yes to the question above, or if he or she had a score of 2 or more in the GDS-4 [45].

### 2.3. Statistical Analyses

Linear regression was used for the multivariable analyses of morale. In the first model, the age groups were included as explanatory factors. In the second model, sociodemographic variables were added. The third model additionally included the social variables and the fourth model the health-related variables. Based on the VIF-value in the collinearity diagnostics, we found that multicollinearity was not a problem.

To test whether possible associations between the sociodemographic, social, and health-related variables and morale varied in the different age groups, joint effects with age were calculated, which correspond to models that include both the main effect and the interaction effect. For example, in the analyses for the joint effects of age and gender on morale 66-year-old men were the reference category for 66-year-old women, 71-year-old men reference category for 71-year-old women, etcetera. The effects were estimated in separate models for each joint effect, adjusting for the main effects of all the other variables.

The analyses were performed using unweighted data. Analyses using weighted data were also tested to ensure that the selection process, i.e., selection of every second person in the city of Vaasa and every third in the city of Umeå and Skellefteå, as opposed to every person in other areas, would not have affected the results. A *p* value of <0.05 was considered statistically significant in the interpretation of the results. The IBM SPSS Statistics version 24 (IBM SPSS Inc., Chicago, IL, USA) was used for all calculations.

## 3. Results

**Figure 1:**
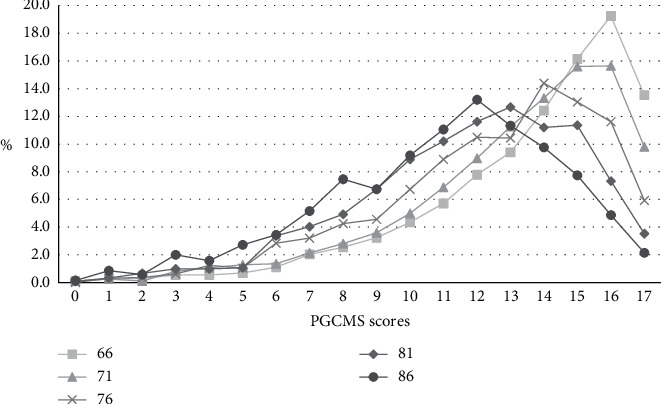
The distribution of scores in the Philadelphia Geriatric Center Morale Scale (PGCMS) in different age groups.

The distribution of PGCMS scores in the different age groups are presented in Figure 1. The results show that the scores in PGCMS are lower in the older age groups (see also Table 2). The characteristics of the sample are presented in Table 2. Regarding the sociodemographic variables, the frequency of most variables were similar across the age groups. There were, however, notable differences regarding having higher educational level and being in a relationship, where the occurrence regarding both variables was lowest in the oldest age group. The proportion of women was also higher in the oldest age group. When looking at the social variables, the frequency of contacts seems to be similar in all age groups whilst the occurrence of high trust in friends and neighbors became lower by the age group. The frequency of having two confidants or more was similar in all age groups, even though a slightly lower prevalence could be noted in the 86-year-olds. The occurrence of perceived loneliness was equally high in the 66- and 71-year-olds (7.9% and 7.6%) but was higher in the older age groups, with the highest prevalence in the oldest age group (18.4%). Regarding the health-related variables, the occurrence of impaired vision, impaired hearing, stroke, pain, poor self-rated health, and depression was higher in the older age groups. The percentage of individuals sleeping well and having mainly own teeth was lower in the older age groups, as well as being independent in IADL and PADL.

The regression analyses are presented in Table 3. In the first model, including only the age groups as explanatory variables for morale, older age was significantly associated with lower morale and this model explained 4.8 percent of the variance in PGCMS. Age remained statistically significant in the other regression models as well, even though the association was notably weaker, especially for the 81- and 86-year-olds, when controlling for the health-related variables (Model 4). Model 2 explained 11 per cent of the variance, Model 3 explained 29 per cent, and Model 4 explained 47 per cent. Based on the *R* square changed, the social variables and the health-related variables explained a proportionately equal share of the variance in PGCMS.

In the final regression model in Table 3 (Model 4), the following variables were associated with lower morale on a statistically significant level (*p* < 0.05): older age, being a woman, perceived loneliness, having gone through a crisis during the preceding year, stroke, pain, poor self-rated health, and depression. Variables associated with higher morale (*p* < 0.05) were making ends meet without difficulties, frequent contacts with children and grandchildren, frequent contacts with friends and neighbors, high trust in friends and neighbors, having two confidants or more, being active in an association, sleeping well, and independence in IADL. Accordingly, all of the social variables remained statistically significant when controlling for the health-related variables. Of the social variables, perceived loneliness and having gone through a crisis during the preceding year had the largest negative association with morale (standardized *β* −0.144 and −0.134). Of the health-related variables, depression had the largest negative association with morale (standardized *β* −0.295) but also poor self-rated health had a larger negative association (standardized *β* −0.180), and sleeping well a larger positive association (standardized *β* 0.154) compared to the other health-related variables that were statistically significant.

Estimates for the joint effects of age and each sociodemographic, social, and health-related variable are presented in Table 4. The associations between morale and perceived loneliness, having gone through a crisis during the preceding year, sleeping well, poor self-rated health, and depression were statistically significant in all age groups. Regarding making ends meet without difficulties, high trust in friends and neighbors, and pain, the associations were statistically significant in all age groups except in the oldest age group. Regarding independence in IADL, the association was statistically significant in the three younger age groups, but not in the two oldest age groups. The association between morale and being a woman, a Swedish-speaker in Ostrobothnia, frequent contacts with children and grandchildren, frequent contacts with friends and neighbors, and stroke also varied in the different age groups but no clear pattern could be distinguished.

## 4. Discussion

The aim of this study was to investigate factors associated with morale in different age groups among old people. The results showed that older age was independently associated with lower morale, but also that the sociodemographic, social, and health-related variables could explain a large proportion of the variance in morale. The analyses including joint effects provided additionally more nuanced information on factors affecting morale in the different age groups. When comparing the regression models, the health-related variables seemed to attenuate the effect of age on morale the most. Nevertheless, considering that all social variables remained statistically significant when controlling for the health-related variables, it is clear that health-related as well as social factors need to be taken into account when promoting morale. Additionally, sociodemographic factors such as gender and perceived economic situation should also be taken into consideration.

In contrast with some previous studies of morale and age [13, 18], the level of morale was lower in the older age groups. The results from the present study are, however, in line with the study of de Guzman et al. [12] and with several studies of measures of SWB, where lower levels of well-being have been noted among the oldest-old (e.g. [20]). The negative association between morale and older age remained when controlling for social and health-related factors, even though the association was weaker. There is no unambiguous explanation to why well-being would be lower in very old age, but mortality-related processes [24] and multimorbidity [25] are two of the possible explanations being discussed. For example, the study by Alcañiz and Solé-Auró [46] showed that the more problems with mobility, discomfort, and emotional distress very old adults have, the more negative impact these factors have on health-related quality of life. Overall, very old adults seem to be at high risk of experiencing adversities [31]. Hence, promoting morale especially among the oldest-old seems to be of importance.

Of the sociodemographic variables included in the study, being a woman was significantly associated with lower morale and making ends meet without difficulties with higher morale, when controlling for social and health-related variables. Being a woman has also in previous studies been associated with lower morale [13, 18], but there are also studies where no differences between men and women have been found (e.g., [12]). It is worth noting that, in contrast to the results of Iwasa et al. [13], the effect of being a woman remained statistically significant also in the multivariable analyses, but interestingly not in all age groups according to the joint effects, highlighting the importance of focusing on the association between morale and gender in future studies. While income previously has been associated with morale [11], our study indicates additionally that the subjective perception of making ends meet without difficulties is associated with higher morale. This implies that subjective as well as objective evaluation of the economic situation is of importance when looking into the life situation of older adults.

In line with previous studies [11, 12], social contacts and social support were associated with morale. Frequent social contacts, having two confidants or more, and having high trust were significantly associated with higher morale, whilst perceived loneliness was associated with lower morale indicating that both quantity and quality of social contacts are of importance. Enabling social contacts into very old age could therefore have important implications for the promotion of morale. It is worth noting that the prevalence of frequent contacts with children and grandchildren as well as with friends and neighbors was similar across all age groups (Table 2). Additionally, more than 60 percent of the sample in all age groups reported having two or more close confidants. These results could be considered to at least partly support the SST. On the one hand, looking at the distribution of these measures, there are no indications of a decrease in social contacts in very old age, which would be in line with the SST. On the other hand, these measures could be considered to represent close social relationships, which older people according to the SST tend to prioritize, corroborating the notion that close relationships are equally important in older age [29]. This also supports the results of Litwin [47], who concluded that older people with a diverse social network including friends and family had higher morale as compared to those with a restricted network. Furthermore, when looking at the joint effects, having frequent contacts with children and grandchildren was significantly associated with higher morale only in the 71- and the 86-year-olds and having frequent contacts with friends and neighbors only in the 66- and the 86-year-olds. Accordingly, there seem to be some differences between the age groups, which could be connected to differences in the nature and function of social networks of younger old and older old [48], but considering that the joint effect does not enable statistical comparisons between the groups, this should be further investigated in order to draw any firm conclusions. Nevertheless, the results showing that both contacts with family and friends are positively associated with morale in the oldest age group are in line with the results of Lara et al. [49], where the oldest-old themselves highlighted the importance of near family and friends as sources for well-being.

Even though the prevalence of frequent social contacts was similar in all age groups, the prevalence of perceived loneliness increased across the age groups. The highest prevalence of loneliness was noted in the oldest age group, which is in line with, for example, the study of Yang and Victor [50]. Corroborating previous notions [51], these results show that the connection between quantity of social contacts and perceived loneliness is not straightforward. It is also important to take into consideration that involuntary social losses are increasingly prevalent with increasing age, in turn inhibiting the possibility to maintain close social contacts which according to SST is important for the well-being of the individual [30]. Of the social variables, perceived loneliness also stands out as the variable with the largest negative association with morale. It was also consistently associated with lower morale on a statistically significant level when looking at the joint effects, indicating that loneliness has a detrimental effect on morale in all age groups. Considering that perceived loneliness also previously has been associated with a decrease in morale over time in very old age [16], interventions targeting loneliness are highly necessary. Importantly, since both social and health-related as well as sociodemographic factors can influence loneliness (e.g., [52]), the underlying causes should be examined in order to be able to provide individually adapted support. Furthermore, since having gone through a crisis in life was associated with lower morale in all age groups, this study support the results of Näsman et al. [17], who highlighted the need of provision of support when a negative life event has occurred in order to prevent a decrease in morale.

As described in the introduction, the association between health and morale, as well as health and SWB, is complex. In the present study, stroke, pain, sleeping well, independence in IADL, poor self-rated health, and depression were independently associated with morale, which corroborates previous findings [11, 18, 53, 54]. Some of the variables including joint effects were significantly associated with morale in all age groups, whilst others were statistically significant only in the younger age groups. Stroke was, for example, associated with lower morale on a statistically significant level only in the youngest age group, and independence in IADL in the three younger age groups but not in the two oldest age groups. These results are intriguing considering that the occurrence of stroke was higher in the older age groups and the number of individuals being independent in IADL was lower. Hence, in line with previous results [16, 32], it might be possible that physical health plays a more important role regarding morale in the younger old than in the older old. Nevertheless, health-related variables are important to take into consideration regardless of age, and associations between these variables and morale in the oldest-old should be further validated, considering that previous research, for example, has found that the oldest-old who have had a stroke had lower morale than those who had not [55]. Furthermore, as stated in the theoretical framework, one explanation to why some individuals seem to be able to experience high levels of well-being despite low physical function could also be connected to the use of SOC-strategies [35]. According to the SOC-theory, long-term well-being is only affected if the individual is not able to compensate for their loss [34]. It is possible that promoting the use of SOC-strategies, i.e., to be more selective, to compensate for losses and to optimize existing personal resources could moderate the negative effect health-related restraints have on morale and could also be beneficial when other crises in life have occurred.

Depression was found to have the largest negative association with morale, which supports previous findings [18], and was associated with lower morale in all age groups. In line with the study on life satisfaction of Puvill et al. [32] and the study on high morale in very old age [16], depression and perceived loneliness seem to be key risk factors of having lower well-being in old age. Considering that self-rated health seems to be closely linked to mental health in old age [56], our results showing that poor self-rated health was negatively associated with morale in all age groups could also serve as a marker of the importance of promoting mental health. Nevertheless, the high prevalence of poor self-rated health especially in the oldest age group (63.7%) calls for further inquiry.

Furthermore, sleeping well was associated with higher morale in all age groups, which is in line with the results of Yokoyama et al. [54]. In their study, both insufficient subjective sleep and the occurrence of different sleep disorders were associated with lower PGCMS scores in a sample of older people. Previous research has also shown that sleep problems and lack of sleep are closely related to quality of life [57], mental well-being [42], and depression [58], and additionally that treating sleep apnea in stroke patients has shown to reduce depressive symptoms [59]. Considering that almost 30 percent of our sample did not sleep well, treating different forms of sleep problems in older people could have important implications regarding the promotion of morale and motivates further exploration.

In sum, factors in all three domains (sociodemographic, social, and health-related) were found to be associated with morale. Regarding age group differences, morale was lower in the older age groups even though fewer variables were significantly associated with morale, especially in the oldest age group compared to the younger. One possible explanation to this could be that the proportion of women in the 86-year-olds was higher, and being a woman was in the regression analyses associated with lower morale, but the analyses including joint effects did not corroborate this interpretation. Another plausible explanation could be found in the sample characteristics (Table 2). When looking at perceived loneliness, poor self-rated health, and depression, which all have a negative association with morale, the occurrence of these is remarkably higher in the older age groups. It seems, thus, that the oldest age groups are more exposed to the main risk factors of having lower morale identified in this study, which in turn could explain why they have a lower level of morale. These findings support the notion that the transition from the third age to the fourth age, often occurring at 80 to 85 years of age, entails extensive challenges to the well-being of the individual [25, 31].

### 4.1. Limitations

This study is based on a representative sample of older people including individuals from age 65 to 86 and contributes to an increased understanding of morale in different stages of old age. However, there are a few points that need to be taken into account when interpreting our results. Considering the cross-sectional design and the inclusion of individuals born from 1930 to 1950, it is impossible to distinguish age and cohort effects. Furthermore, there is a risk of nonresponse bias in all age groups, especially in the 86-year-olds who had the lowest response rate. It could, for example, be expected that the included sample is to some extent healthier than the overall oldest-old population, considering that factors such as higher levels of multimorbidity and frailty compared to younger age groups are likely to affect the probability of participating in a survey. Nevertheless, for the same reasons, it could be expected that the actual occurrence of, for example, poor self-rated and depression would be even higher among the oldest-old, implying that the conclusions drawn regarding these risk factors would still be valid. The sample sizes in the older age groups were also smaller, due to fewer oldest-old adults within the population. Hence, it cannot be ruled out that the results regarding that some variables were statistically significant only in the younger age groups were affected by differences in the sample sizes. Due to missing values in variables included in the regression analyses, a part of the sample was also excluded from the final regression model. There is also a possibility that factors previously associated with morale, which were not available in our data, such as personality [9] and cognitive function [17], would have affected the results.

## 5. Conclusions

Based on the results, a comprehensive view of the living conditions of the individual is needed in order to successfully promote morale in old age. It is evident that sociodemographic, social, and health-related variables are important to take into account when assessing risk factors and promoting factors for morale. Perceived loneliness, having gone through a crisis in life, poor self-rated health, and depression were associated with lower morale, whilst sleeping well was associated with higher morale, in all age groups. Furthermore, independence in IADL was associated with lower morale only in the three younger age groups, suggesting that physical function played a more important role in younger old than in the oldest age groups. Nevertheless, it is worth noting that especially the oldest age groups seem to be exposed to several risk factors of lower morale identified in this study. Multidimensional social and health interventions targeting especially mental health and social inclusion among the oldest-old could therefore be recommended. Future research could examine whether the use of SOC-strategies could be beneficial when strengthening morale.

## Figures and Tables

**Table 1:** Number of participants and response rates in different regions and age groups (*n* = 9386).

Sample	*n*	Response rate %
Region		
Västerbotten, Sweden	4375	70.8
Swedish-speakers in Ostrobothnia, Finland	2296	61.7
Finnish-speakers in Ostrobothnia and Southern Ostrobothnia, Finland	2715	54.9
Age group^a^		
66-year-olds	2751	62.5
71-year-olds	2863	67.2
76-year-olds	1692	64.7
81-year-olds	1300	59.1
86-year-olds	759	50.3
Total	9386	63.3

^a^21 respondents had a missing value in the age group variable.

**Table 2:** Sample characteristics according to age group and of the total sample (*n* = 9047).

	66-year-olds (*n* = 2711) *M* (SD) (%)	71-year-olds (*n* = 2806) *M* (SD) (%)	76-year-olds (*n* = 1619) *M* (SD) (%)	81-year-olds (*n* = 1214) *M* (SD) (%)	86-year-olds (*n* = 697) *M* (SD) (%)	Total (*n* = 9047) *M* (SD) (%)
PGCMS (continuous)	13.6 (3.1)	13.1 (3.2)	12.4 (3.3)	11.7 (3.3)	10.9 (3.4)	12.8 (3.3)
Woman	53.3	52.1	51.7	54.1	63.4	53.5
Higher educational level	72.7	60.5	50.9	46.7	39.1	59.0
In a relationship	82.1	79.5	72.1	62.2	44.3	74.0
Region						
Västerbotten, Sweden	43.8	47.0	51.4	46.7	46.9	46.7
Swedish-speakers in Ostrobothnia, Finland	23.1	25.6	22.1	25.3	27.4	24.3
Finnish-speakers in Ostrobothnia and Southern Ostrobothnia, Finland	33.1	27.4	26.6	28.0	25.7	28.9
Place of residence						
Urban	40.2	40.7	41.7	43.7	41.7	41.2
Semiurban	18.3	19.1	22.4	19.1	22.5	19.7
Rural	41.4	40.2	35.9	37.2	35.8	39.1
Born in the same place as you live now	36.6	33.6	33.1	36.0	33.7	34.7
Making ends meet without difficulties	63.3	65.1	64.0	64.6	67.1	64.4
Frequent contacts with children and grandchildren	56.1	56.0	51.1	54.4	55.3	54.9
Frequent contacts with friends and neighbors	40.8	44.2	42.8	42.3	41.1	42.4
High trust in friends and neighbors	68.3	66.3	64.5	58.5	56.0	64.8
2 confidants or more	69.9	68.5	66.3	67.7	60.3	67.8
Active in an association	45.3	48.1	52.6	47.6	39.7	47.4
Perceived loneliness	7.9	7.6	10.5	14.4	18.4	9.9
Gone through a crisis during the preceding year	46.6	43.9	46.2	47.5	45.8	45.7
Impaired vision	0.9	1.5	1.8	2.2	4.2	1.7
Impaired hearing	0.7	1.2	1.5	2.4	5.3	1.6
Stroke	4.4	6.7	9.3	11.4	13.0	7.5
Pain	47.2	47.1	46.3	51.1	55.4	48.1
Sleeping well	73.1	70.5	70.4	68.2	63.1	70.4
Mainly own teeth	85.9	77.4	68.7	59.6	48.4	74.1
Independent in IADL	76.2	66.3	53.1	38.2	28.7	60.2
Independent in PADL	96.2	95.3	93.6	92.2	85.2	94.1
Poor self-rated health	23.9	30.7	40.5	52.0	63.7	35.7
Depression	10.7	11.8	13.7	15.1	22.4	13.0

*Note*. Percentages are reported for dichotomous variables, and mean (*M*) and standard deviation (SD) for continuous variables. PGCMS: Philadelphia Geriatric Center Morale Scale; IADL: Instrumental Activities of Daily Living; PADL: Personal Activities of Daily Living.

**Table 3:** Estimated effects of age and sociodemographic, social, and health-related factors on morale according to linear regression models (*n* = 6460).

Variable	Categories	Model 1	Model 2	Model 3	Model 4
		Standardized estimate	Standardized estimate	Standardized estimate	Standardized estimate
Age group	66-year-olds^a^				
	71-year-olds	−0.065^*∗∗∗*^	−0.066^*∗∗∗*^	−0.073^*∗∗∗*^	−0.046^*∗∗∗*^
	76-year-olds	−0.129^*∗∗∗*^	−0.125^*∗∗∗*^	−0.120^*∗∗∗*^	−0.078^*∗∗∗*^
	81-year-olds	−0.170^*∗∗∗*^	−0.165^*∗∗∗*^	−0.141^*∗∗∗*^	−0.088^*∗∗∗*^
	86-year-olds	−0.174^*∗∗∗*^	−0.165^*∗∗∗*^	−0.135^*∗∗∗*^	−0.075^*∗∗∗*^
Gender	Man^a^				
	Woman		−0.072^*∗∗∗*^	−0.066^*∗∗∗*^	−0.046^*∗∗∗*^
Educational level	Lower^a^				
	Higher		0.006	0.002	−0.007
Marital status	Single^a^				
	In a relationship		0.048^*∗∗∗*^	−0.010	0.004
Region	Västerbotten, Sweden^a^				
	Swedish-speakers in Ostrobothnia, Finland		−0.015	−0.016	−0.015
	Finnish-speakers in Ostrobothnia and Southern Ostrobothnia, Finland		0.003	0.022	0.002
Place of residence	Urban^a^				
	Semiurban		−0.018	−0.015	−0.004
	Rural		0.009	0.005	0.001
Born in the same place as you live now	No^a^				
	Yes		0.022	0.009	0.018
Making ends meet	With difficulties^a^				
	Without difficulties		0.217^*∗∗∗*^	0.153^*∗∗∗*^	0.093^*∗∗∗*^
Contacts with children and grandchildren	Less frequent^a^				
	Frequent			0.043^*∗∗∗*^	0.032^*∗∗∗*^
Contacts with friends and neighbors	Less frequent^a^				
	Frequent			0.033^*∗∗∗*^	0.026^*∗∗*^
Trust in friends and neighbors	Low^a^				
	High			0.114^*∗∗∗*^	0.068^*∗∗∗*^
Number of confidants	0-1^a^				
	2 or more			0.043^*∗∗∗*^	0.021^*∗*^
Active membership in an association	None or passive^a^				
	1 or more			0.076^*∗∗∗*^	0.034^*∗∗∗*^
Perceived loneliness	No^a^				
	Yes			−0.305^*∗∗∗*^	−0.144^*∗∗∗*^
Gone through a crisis during the preceding year	No^a^				
	Yes			−0.221^*∗∗∗*^	−0.134^*∗∗∗*^
Impaired vision	No^a^				
	Yes				0.001
Impaired hearing	No^a^				
	Yes				−0.018
Stroke	No^a^				
	Yes				−0.029^*∗∗*^
Pain	No^a^				
	Yes				−0.057^*∗∗∗*^
Sleeping well	No^a^				
	Yes				0.154^*∗∗∗*^
Own teeth	No^a^				
	Yes				−0.013
Independent in IADL	No^a^				
	Yes				0.063^*∗∗∗*^
Independent in PADL	No^a^				
	Yes				−0.006
Self-rated health	High^a^				
	Poor				−0.180^*∗∗∗*^
Depression	No^a^				
	Yes				−0.295^*∗∗∗*^
Adjusted *R* square		0.048	0.108	0.297	0.473
*R* square changed			0.061	0.189	0.176

*Note*. Standardized betas are reported. Model 1 describes the association between age and PGCMS scores using dummy variables for each age group. Model 2 includes additionally sociodemographic variables. Model 3 includes also the social variables, and Model 4 includes additionally the health-related variables. ^a^Reference category. PGCMS: Philadelphia Geriatric Center Morale Scale; IADL: Instrumental Activities of Daily Living; PADL: Personal Activities of Daily Living. ^*∗∗∗*^*p* < 0.001, ^*∗∗*^*p* < 0.01, and ^*∗*^*p* < 0.05.

**Table 4:** Estimated joint effects of age and different sociodemographic, social, and health-related variables on morale (*n* = 6460).

Variable	Categories	66-year-olds	71-year-olds	76-year-olds	81-year-olds	86-year-olds
		Standardized estimate	Standardized estimate	Standardized estimate	Standardized estimate	Standardized estimate
Gender	Man^a^					
	Woman	−0.041^*∗∗*^	−0.025^*∗*^	−0.022	−0.041^*∗∗*^	−0.007
Educational level	Lower^a^					
	Higher	−0.008	−0.021	0.008	−0.003	0.011
Marital status	Single^a^					
	In a relationship	0.025	−0.009	−0.017	0.024	−0.009
Region	Västerbotten, Sweden^a^					
	Swedish-speakers in Ostrobothnia, Finland	−0.021^*∗*^	−0.013	0.009	−0.004	0.017
	Finnish-speakers in Ostrobothnia and Southern Ostrobothnia, Finland	−0.001	−0.002	0.013	−0.007	0.000
Place of residence	Urban^a^					
	Semiurban	−0.005	0.003	−0.004	0.002	−0.007
	Rural	−0.017	0.009	0.009	0.001	0.012
Born in the same place as you live now	No^a^					
	Yes	0.006	0.018	0.009	0.010	−0.002
Making ends meet	With difficulties^a^					
	Without difficulties	0.084^*∗∗∗*^	0.075^*∗∗∗*^	0.050^*∗∗*^	0.086^*∗∗∗*^	0.019
Contacts with children and grandchildren	Less frequent^a^					
	Frequent	0.023	0.029^*∗*^	0.007	0.016	0.027^*∗*^
Contacts with friends and neighbors	Less frequent^a^					
	Frequent	0.039^*∗∗∗*^	0.018	−0.001	−0.021	0.023^*∗*^
Trust in friends and neighbors	Low^a^					
	High	0.042^*∗∗*^	0.073^*∗∗∗*^	0.054^*∗∗∗*^	0.032^*∗*^	0.024
Number of confidants	0-1^a^					
	2 or more	0.025	0.023	0.008	0.015	0.003
Active membership in an association	None or passive^a^					
	1 or more	0.015	0.022	0.047^*∗∗∗*^	0.011	−0.002
Perceived loneliness	No^a^					
	Yes	−0.095^*∗∗∗*^	−0.075^*∗∗∗*^	−0.063^*∗∗∗*^	−0.061^*∗∗∗*^	−0.026^*∗*^
Gone through a crisis during the preceding year	No^a^					
	Yes	−0.096^*∗∗∗*^	−0.105^*∗∗∗*^	−0.063^*∗∗∗*^	−0.060^*∗∗∗*^	−0.033^*∗∗*^
Impaired vision	No^a^					
	Yes	−0.010	0.000	0.001	0.010	0.003
Impaired hearing	No^a^					
	Yes	−0.008	−0.004	−0.011	−0.009	−0.010
Stroke	No^a^					
	Yes	−0.019^*∗*^	−0.011	−0.014	−0.011	−0.014
Pain	No^a^					
	Yes	−0.043^*∗∗∗*^	−0.029^*∗*^	−0.027^*∗*^	−0.052^*∗∗∗*^	−0.021
Sleeping well	No^a^					
	Yes	0.151^*∗∗∗*^	0.119^*∗∗∗*^	0.124^*∗∗∗*^	0.124^*∗∗∗*^	0.039^*∗*^
Own teeth	No^a^					
	Yes	0.002	−0.021	−0.003	−0.010	−0.015
Independent in IADL	No^a^					
	Yes	0.072^*∗∗∗*^	0.052^*∗∗∗*^	0.036^*∗∗*^	0.017	0.019
Independent in PADL	No^a^					
	Yes	−0.032	−0.015	0.010	0.015	−0.022
Self-rated health	High^a^					
	Poor	−0.116^*∗∗∗*^	−0.108^*∗∗∗*^	−0.094^*∗∗∗*^	−0.078^*∗∗∗*^	−0.057^*∗∗∗*^
Depression	No^a^					
	Yes	−0.169^*∗∗∗*^	−0.178^*∗∗∗*^	−0.120^*∗∗∗*^	−0.114^*∗∗∗*^	−0.093^*∗∗∗*^

*Note*. Standardized betas are reported. Estimates for the joint effect of age group and each explanatory variable are presented row-wise in the table. Separate models were calculated for each joint effect, while adjusting for the main effects of all the other explanatory variables. The joint effects were estimated within the age groups, not between them. The estimates for each joint effect were calculated by switching the reference categories, meaning that the row-wise values reported are in relation to reference categories within each age group, i.e., 66-year-old men are, for example, the reference category to 66-year-old women. ^a^ Reference category. PGCMS: Philadelphia Geriatric Center Morale Scale; IADL: Instrumental Activities of Daily Living; PADL: Personal Activities of Daily Living. ^*∗∗∗*^*p* < 0.001, ^*∗∗*^*p* < 0.01, and ^*∗*^*p* < 0.05.

## Data Availability

The GERDA survey data used to support the findings of this study may be released upon application to the GERDA steering committee who can be contacted at fredrica.nyqvist@abo.fi.
